# Choosing to biopsy or refer suspicious melanocytic lesions in general practice

**DOI:** 10.1186/1471-2296-13-78

**Published:** 2012-08-08

**Authors:** Sean Robison, Marjan Kljakovic, Peter Barry

**Affiliations:** 1 MBBS (Hons), Medical School, Australian National University, Canberra, 0200, ACT, Australia; 2 FRACGP, PhD, Professor of General Practice, Academic Unit of General Practice and Community Health, Australian National University Medical School, Canberra, 0200, ACT, Australia; 3 MBBS, FRACS, Surgical Oncologist, Canberra Melanoma Unit, Calvary Clinic, Canberra, 2617, ACT, Australia

**Keywords:** Biopsy, General practice, Guidelines, Melanoma, Audit

## Abstract

**Background:**

General practitioners (GPs) are involved in the management of most melanocytic skin lesions in Australia. A high quality biopsy technique is a crucial first step in management, as it is recognized that poor techniques can mislead, delay, or miss a diagnosis of melanoma. There has been little published on the biopsy decisions and techniques of GPs. This study aims to describe the current management choices made by GPs for suspicious melanocytic skin lesions and to compare their choices with the best practice guidelines.

**Methods:**

An anonymous survey of GPs presented with three clinical scenarios with increasing complexity of melanoma in which a referral or biopsy decision was specified.

**Results:**

391 mailed surveys with a 76.3% response rate. Mean biopsy experience was 4.14 biopsies per GP per month. The rates of choosing to refer among the three scenarios were 31%, 52% and 81% respectively, with referral to surgery being the most common choice (81%). Most biopsy techniques (55%) were chosen according to best practice guidelines, although non-guideline biopsy techniques chosen included shave (n = 10), punch biopsy (n = 57), wide excisions (n = 65), and flaps (n = 10). The few GPs (n = 5) who identified themselves as skin specialist GPs were no more likely to adhere to guidelines than their colleagues.

**Conclusion:**

A majority of referrals and biopsies were chosen by GPs according to best practice guidelines, but concern remains for the high proportion of GPs making non-guideline based choices. How GPs choose to biopsy or refer needs further training, audit, and research if Australia is to improve the outcome of melanoma management in general practice.

## Background

Melanoma remains the most common cancer in the 30-51 year old age group and accounts for 3.9% of all cancer deaths in Australia [1]. The current 5 year survival rate is 92%, a statistic which relies in part on the early detection and accurate diagnosis of suspicious melanocytic lesions [1].

The general practitioner (GP) is the first point of medical contact for the majority of patients who have suspicious skin lesions in Australia [2,3]. Previous research has explored the initial step in the diagnostic process – the assessment of skin lesions presented by patients [4,5]. The next step is to choose either to biopsy the lesion or to refer. A biopsy of melanocytic skin lesions is crucial as pathological determination of tumour depth influences management and prognosis. Furthermore, poor biopsy techniques can mislead, delay, or miss a diagnosis of melanoma [6]. A few studies have reviewed biopsy margins achieved by GPs [7-9], but none have examined how GPs choose to refer or biopsy when presented with suspicious melanocytic lesions.

The management of melanoma has been outlined in recommended guidelines. Commonly accessed sources of guidelines are the Australian National Health and Medical Research Council (NHMRC) melanoma guidelines [10]. These guidelines have specific comments on when doctors should biopsy and when to refer for a range of melanoma sub-types. Retrospective reviews of the histopathology and biopsy type of confirmed melanomas have focused on the clinical practice of predominantly dermatologists and surgeons [8,11,12], rather than focusing on GPs.

This study seeks to describe how GPs choose to biopsy or refer suspicious melanocytic skin lesions, and to compare their choices with best practice guidelines.

## Methods

### Subjects

In September 2008 a questionnaire was sent to 391 GPs from the urban Australian Capital Territory (ACT) region.

### Questionnaire design

The anonymous questionnaire consisted of three clinical scenarios of increasing complexity of suspicious melanocytic skin lesions and closed questions about the GPs’ choice to refer or biopsy in each scenario as shown in Table 1.

**Table 1:** Scenarios

	
**SCENARIO 1**	On routine examination you notice a 1cm suspicious pigmented melanocytic lesion on a patient’s mid-back. You are concerned about the possibility of melanoma. There is plenty of redundant skin in the area.
**SCENARIO 2**	A patient complains to you about an itchy lesion on the ankle. There is a 5mm lesion, which you think may be melanoma. You judge complete removal of the lesion with primary closure just possible. Foot pulses are present.
**SCENARIO 3**	A patient claims that a large area of pigmentation on her cheek has recently changed colour. Clinically she has a Hutchinson’s melanotic freckle. You think it may be a melanoma. It measures 2 x 1cm and complete removal would probably need a flap.
FOR EACH SCENARIO:* Question 1 - what is your next action? *Question 2 - if you chose to refer, where would you refer?* *Questions 3 - if you do the biopsy yourself, please indicate your technique?*	

*The list of closed options for biopsy techniques are detailed in results section Table 2.

**Table 2:** Referrals and biopsy techniques chosen by 285 general practitioners for three clinical scenarios of patients presenting with suspicious melanocytic lesion

**Response**	**Scenario 1**		**Scenario 2**		**Scenario 3**	
Total Number	285		285		285	
Missing choice data	1		0		17	
GP chooses* to refer	89	31%	147	52%	231	81%
Another GP	30	31%	30	18%	13	6%
A dermatologist	22	21%	34	20%	35	15%
A general surgeon	24	25%	55	32%	37	16%
A plastics surgeon	22	21%	51	30%	151	64%
Missing referral data	10		10		19	
GP chooses to biopsy	195	68%	138	48%	37	14%
A shave biopsy	2	1%	3	2%	5	14%
An incisional punch biopsy (2-4mm diameter)	17	9%	18	13%	22	61%
An incisional biopsy	1	0.5%	3	2%	5	14%
An excision biopsy 1-2 mm margins**	97	50%	72	53%	NA	
An excision biopsy 5-10 mm margins	52	27%	13	9%	NA	
An excision biopsy (1-2 mm margin) with primary closure	NA		NA		1	3%
An excision biopsy (1-2 mm margin) with a flap	NA		3	2%	4	11%
Other	1	0.5%	1	0.5%	0	0%
Missing biopsy data	25	13%	25	18%	0	0%
Adheres to guidelines#	186	65%	219	77%	231	86%

* GPs could choose more than one kind of referral.

** Biopsy technique suggested in 1999 Guideline.

NA – Indicates that choice of biopsy technique not given in the scenario.

# Combined referral and 1999 Guideline.

In each of the scenarios, it was clearly stated that melanoma was the provisional diagnosis. A lesion description, rather than a photo, was employed because the study sought a choice of management action instead of focusing on the clinical diagnosis of a specific skin lesion.

In each scenario, the first question asked if the GP would refer or perform a biopsy. The second question asked to whom the referral would be made. The third question asked which kind of biopsy technique would be chosen (biopsy options were indicated and other suggestions optional). Questions on GPs’ work practices and whether they had any formal training in dermoscopy or skin biopsy techniques were also asked.

Results were analysed using SPSS and, where appropriate, the Chi squared ( [2] test compared differences between categories. A content analysis was done of the responses to the open-ended questions in order to identify themes. There was one reminder letter sent, and then one month later the remaining non-responding GPs were sent a reminder fax.

### Adherence to guidelines

The NHMRC clinical practice guidelines for the management of melanoma were used as the benchmark for classifying whether a GP adhered to guidelines for each scenario [10]. The 1999 version of the guidelines were used, as the study period concluded at the time of the 2008 updated guideline release [13]. In order to limit potential response bias, the GPs were not asked if they were aware of the NHMRC guidelines. The list of eight possible biopsy types is shown in Table 2. In the first two scenarios, a GP was classified as having adhered to the guideline if the GP chose to refer or to perform an excision biopsy with 1-2 mm margins. In the third scenario, a GP was classified as having adhered to the guideline if, and only if, the GP chose to refer.

Ethical approval was obtained from the Australian National University Human Ethics Committee and the ACT Health Human Research Ethics Committee.

## Results

### Response

Of the 391 people contacted, 299 responded to the survey (76.3%) of which 285 (72.7%) were included for data analysis. Excluded responses included retired GPs or those not located at a general practice. Among the responders, 87 GPs (29%) worked part-time in general practice, and 20 GPs (7%) worked in a specialized area of general practice, of which five worked in skin cancer management.

### Clinical experience

The reported mean number (SD) of biopsies performed in clinical practice by 239 GPs was 4.14 (8.65) biopsies per month. The range was 0 to 70 biopsies per month with 221 GPs (92.5%) performing up to 10 biopsies per month, and 56 GPs (20%) reported none. Fewer GPs (142, 48%) reported routine use of dermoscopy in clinical practice. The five GPs who specialized in skin cancer management performed a mean of 44.80 (17.68) biopsies per month, and all routinely used dermoscopy.

### Training

More GPs (132, 44%) reported receiving formal training in skin biopsy techniques, than formal training in dermoscopy (81, 27%). There was no difference in the proportion of part-time GPs who had formal training in dermoscopy compared to full-time GPs (p = 0.278), however, significantly fewer part-time GPs had formal training in biopsy techniques compared to full-time GPs (42% versus 62%, χ^2^ test = 8.175, df =1, p = 0.004). All five GPs who specialised in skin cancer management had formal training in both dermoscopy and biopsy techniques.

### The choice to refer

There were 285 GPs who chose to refer, among whom 83 GPs (29%) referred in all three scenarios, 61 GPs (21%) referred in two scenarios, 94 GPs (33%) referred in only one scenario, and 45 GPs (16%) referred none. Table 2 shows that the referral rates for the three scenarios were 31%, 52%, and 81% respectively. More part-time GPs referred in one or more scenarios compared to full-time GPs (93% versus 85%, χ^2^ test = 16.503, df =3, p = 0.001), and similarly, more GPs without training in biopsy techniques referred in one or more scenarios compared to GPs who did have training (97% versus 81%, χ^2^ test = 22.555, df =3, p = 0.0001). None of the five GPs who specialised in skin cancer management referred in any scenario.

There were 428 instances where referral to a specialist was chosen. In 224 instances (44%) referrals were made to plastics surgeons, 116 referrals (23%) to general surgeons, 91 referrals (18%) to dermatologists, and 73 referrals (15%) to other GPs who did biopsies.

There were 239 GPs who indicated they would refer in one or more scenario, among whom 52 GPs (18%) referred to more than one kind of specialist. There were 49 GPs who referred at least one scenario to another GP and 55 GPs had another GP within their general practice who performed biopsies. More GPs referred patients to another GP if there was a GP within the practice who performed biopsies compared to practices that did not have another GP who performed biopsies (50% versus 30%, χ^2^ test = 4.043, df =1, p = 0.044).

### The choice of biopsy technique

Table 2 shows the biopsy rates for the scenarios were 68%, 48%, and 14% respectively. There were 29 GPs (10%) who chose to biopsy in all three scenarios, 111 GPs (39%) chose to biopsy in two scenarios, 61 GPs (21%) chose to biopsy in one scenario only, and 84 GPs (30%) chose to biopsy in none. More full-time GPs chose to biopsy in one or more scenario compared to part-time GPs (77% versus 61%, χ^2^ test = 17.460, df =3, p = 0.001). More GPs with training in biopsy techniques chose to biopsy in one or more scenarios compared to GPs who did not have training (80% versus 60%, χ^2^ test = 21.606, df =3, p = 0.0001). All five GPs who specialised in skin cancer management chose to biopsy in all three scenarios.

Table 3 shows GP’s comments reporting why they chose not to perform a biopsy in one or more of the scenarios. There was no significant variation in these opinions for part-time GPs and GPs who did not have training in biopsy techniques.

**Table 3:** Reasons why 156 general practitioners did not perform a biopsy in one or more scenarios of patients presenting with suspicious melanocytic lesions

**Reason for not performing a biopsy**	**Positive response**	**%**
You do not feel comfortable doing the biopsy	101	60%
You have no interest in performing any biopsies	21	13%
Your surgery is not equipped for this	8	5%
Other comments:	38	23%
Qualitative analysis of other specified comments*:		
*Biopsy requires an expert*		
“If it is melanoma it needs specialist care”		
“It may not need a biopsy if a dermatologist looks at it”		
“The other GP in our practice does biopsies”		
*Difficulty with biopsy technique*		
“Clinical melanoma needs a wide local excision”		
“I prefer not to excise cosmetically sensitive areas”		
*Personal issues*		
“I have arthritic fingers”		
“I do not excise from the face”		
*Systems issues*		
“Doing biopsy is time consuming - not well remunerated”		
“There are medico legal risks with doing facial lesions”		
“There is more co-coordinated care for referrals”		

*GP could have more than one comment.

### Adherence to the 1999 clinical practice guidelines for the management of melanoma

The correct choice recommended by the guidelines to perform “An excision biopsy 1-2 mm margins” was taken in 169 instances (55%). Table 2 shows the adherence rates to guidelines for each of the three scenarios were 65%, 77%, and 86% respectively.

In the first scenario, there was no significant relationship between adherence to guidelines and formal training in dermoscopy (p = 0.532). In the second scenario, more GPs with formal training in dermoscopy adhered to guidelines compared to GPs who did not adhere (49% versus 27% χ^2^ test = 5.279, df =1, p = 0.022). In the third scenario, more GPs with formal training in dermoscopy did not adhere to guidelines compared to GPs who did adhere (71% versus 40% χ^2^ test = 9.814, df = 1, p = 0.002).

In the first two scenarios, there was no significant relationship between adherence to guidelines and formal training in biopsy techniques (p = 0.199 and p = 0.354 respectively). In the third scenario more GPs with formal training in biopsy techniques did not adhere to guidelines compared to GPs who did not have training (86% versus 49%, χ^2^ test = 16.019, df =1, p = 0.000).

Completion of all three scenarios was done by 267 GPs, among whom 153 GPs (54%) adhered to guidelines in all three scenarios, 70 GPs (25%) adhered in two scenarios, 33 GPs (12%) adhered in one scenario, and 11 GPs (4%) adhered in none. Table 4 shows the characteristics of GPs and the number of scenarios where GPs demonstrated adherence to guidelines. More GPs demonstrated adherence in none and two scenarios if they had any training (p = 0.001), routinely used dermoscopy (p = 0.002), and had a higher clinical biopsy rate (p = 0.001). Paradoxically, fewer GPs demonstrated adherence in one and three scenarios for these characteristics.

**Table 4:** The characteristics of GPs and their adherence to guidelines for up to three scenarios of patients presenting with suspicious melanocytic lesions

**GP characteristic**	**Number of GPs**	**Number of scenarios where GPs adhered to guidelines**							
		**None of the Scenarios**		**One Scenario**		**Two Scenarios**		**Three Scenarios**	
Training*									
Dermoscopy only	19	0	0%	1	5%	7	37%	11	58%
Biopsy only	69	4	6%	6	9%	25	36%	34	49%
Trained in both	62	5	8%	5	8%	21	34%	31	50%
No training	94	1	1%	11	12%	17	18%	65	69%
Full-time**	137	6	4%	18	13%	38	28%	75	56%
Part-time	86	3	3%	5	6%	22	22%	56	56%
GP specialises in skin cancer management	5	1		0		4		0	
Routine use of dermoscopy#		7	78%	13	57%	52	74%	62	49%
Mean (SD) biopsies per month##		8.50	(8.11)	3.30	(3.47)	8.21	(14.37)	1.98	(2.86)

*(n = 242 GPs, χ^2^ test = 43.238, df =12, p = 0.000).

**(Mann-Whitney U test, n = 242 GPs, z = -1.72, p = 0.085).

# (Mann-Whitney U test, n = 223 GPs, z = -3.076, p = 0.002).

## (One-way ANOVA, n = 237 GPs, df = 3, F = 9.657, p < 0.01).

## Discussion

This study demonstrates that a majority of GPs in the ACT adhere to best practice guidelines for suspicious melanocytic lesions by choosing to refer for a specialist opinion where appropriate (in 54% of instances) – the safest option suggested by best practice guidelines in 1999 [10]. In this study, most referrals were to general surgeons, plastic surgeons and dermatologists. However, 17% of referrals were to other GPs, particularly if the GP who performed biopsies worked within the referring GP’s general practice. In 18% of scenarios, GPs chose to refer cases to more than one specialist. Comments from these respondents indicated a frustration with extended waiting times for specialist care, and a need for multiple referrals in order to maximize the chances of an earlier assessment. Although this study reflects the availability of local services in the ACT region, specialists in general may acknowledge potential access issues and consider more streamlined approaches to assessment and management.

The majority of GPs (84%) chose to perform a biopsy in one or more scenarios and most GPs (55%) chose the guideline recommended biopsy technique of an excision biopsy with 1-2 mm margin for scenarios one and two. Concern exists for the remaining non-guideline biopsy techniques chosen because of the inherent risks associated with them. In this study, there were 66 scenarios (21% of all choices to biopsy) where incisional or punch biopsies were chosen. The risks associated with these techniques include sampling error, missing active invasive melanoma, and creating an area of fibrosis in the remaining tissue, which may confuse the pathology in further excisions [9,11,14]. There were 65 instances (21%) where large diameter biopsies were chosen. This technique would obtain a sufficient margin around a melanotic lesion, but may result in unnecessary cosmetic disfigurement – an important element considering the number needed to treat (NNT) melanoma ranges from 11 to 83 depending on patient and doctor characteristics [15]. A wider margin could also compromise further excision and alter the lymphatic drainage, potentially impacting on future sentinel node mapping. There were 10 scenarios (3%) where shave biopsies were chosen, a technique that has been shown to poorly identify tumour depth, limiting accurate staging of the melanoma lesion [11]. Shave biopsy may sample an area of regression where melanoma has invaded below the shave, or an area of invasion that is clinically inconspicuous [11,16]. Furthermore, a recent study in Australia has highlighted the increased rates of misdiagnosed melanoma histopathology with the use of shave and punch biopsies, when compared to excisional biopsy [17]. Finally, in seven scenarios (2%) skin flaps were chosen with excision biopsies. Researchers have identified an increasing trend in general practice towards the use of flaps, particularly in skin cancer clinics [3]. Performing flaps without a histological confirmation of margins can confuse re-excision because the remaining margins have been altered, requiring more extensive and complex definitive excision. Non-guideline techniques may ultimately compromise a timely diagnosis.

Characteristics of the GP had a profound impact on both the decision to refer and the choice of biopsy technique. Part-time GPs were less likely to train in dermoscopy or biopsy techniques and were more likely to refer than their full-time colleagues. Conversely, GPs who worked full-time had more experience of biopsy in their clinical practices and were more likely to choose to biopsy in the scenarios. Finally, the few GPs who identified themselves as specialists in skin cancer management did not refer any cases to other specialists and performed four times more biopsies per month than their colleagues - yet paradoxically they were no more likely to adhere to guidelines than their colleagues.

The frequent use of non-guideline biopsy techniques in this study supports the argument for further training of GPs in melanoma management. Numerous studies recognize that targeted training has a variable impact on identification and NNT of melanoma [4,18,19]. This study confirms the mixed effect on guideline adherence following training, suggesting that the type of training done in the past may not be sufficient. For example, rather than GPs train by going to formal courses away from their place of work, perhaps improved feedback mechanisms at the time of biopsy may improve adherence to guidelines. This feedback could come in the form of regular clinical audit of the GP’s pathology results and their adherence with guidelines. An example of a current audit system is the Skin Cancer College of Australia and New Zealand Skin Cancer Audit Research Database (http://www.skincanceraudit.com). Future research on how GPs might improve adherence to guidelines is needed.

This study was limited in that we provided only three written scenarios, and analyzed the actions GPs stated they would perform in a theoretical context, rather than analyse their actual clinical behavior. The use of the term ‘may’ in the scenarios could have introduced ambiguity in the strength of GP responses. The referral patterns we observed may have reflected the availability of local surgical services. This study did not assess the influence of private health insurance on GP decisions to refer or biopsy. The low rate of adherence in scenario three might reflect the change over time in the guideline advice from generalized to specialized approaches in the management of lentigo maligna melanoma (LMM) [10]. This study supports the need for a more standardized approach to LMM management in general practice. Another consideration is that the circulation of the draft 2008 NHMRC melanoma guidelines coincided with the conclusion of our study and may have biased a limited number of late responders [13].

## Conclusion

A majority of referrals and biopsies were chosen by GPs according to best practice guidelines, but concern remains for the high proportion of GPs making non-guideline based choices. Further training, audit, and research on how GPs choose to biopsy or refer needs to be considered if Australia is to improve the outcome of melanoma management in general practice.

## Competing interests

The authors declare that they have no competing interests.

## Authors’ contributions

SR contributed to study design, collected, analysed and interpreted the data, and drafted the manuscript. MK contributed with funding, data analysis and interpretation, content and critical review of the manuscript. PB conceived the study idea, contributed funding, and critically reviewed the manuscript. All authors read and approved the final document.

## Pre-publication history

The pre-publication history for this paper can be accessed here:

http://www.biomedcentral.com/1471-2296/13/78/prepub
